# Rab27b Is a Potential Predictor for Metastasis and Prognosis in Colorectal Cancer

**DOI:** 10.1155/2014/913106

**Published:** 2014-12-16

**Authors:** Jun Bao, Yijiang Ni, Hui Qin, Li Xu, Zhijun Ge, Feng Zhan, Huijun Zhu, Jiabi Zhao, Xiaoli Zhou, Xiaojun Tang, Liming Tang

**Affiliations:** ^1^Department of Gastroenterology, Jiangsu Province Geriatric Hospital, Nanjing 210000, China; ^2^Department of Traumatic Surgery, Changzhou No. 2 People's Hospital Affiliated to Nanjing Medical University, Changzhou 213000, China; ^3^Department of Intensive Care Medicine, Changzhou No. 2 People's Hospital Affiliated to Nanjing Medical University, Changzhou 213000, China; ^4^Department of Pathology, Jiangsu Cancer Hospital, Affiliated Cancer Hospital of Nanjing Medical University, Nanjing 210000, China; ^5^Department of Anesthesiology, Yixing People's Hospital, The Affiliated Yixing Hospital of Jiangsu University, Yixing 214200, China; ^6^Department of Hepatobiliary and Laparoscopic Surgery, Yixing People's Hospital, The Affiliated Yixing Hospital of Jiangsu University, Yixing 214200, China; ^7^Department of Pathology, Affiliated Hospital of Nantong University, Nantong 226000, China; ^8^Department of Pathology, Changzhou No. 2 People's Hospital Affiliated to Nanjing Medical University, Changzhou 213000, China; ^9^The Key Laboratory of Cancer Biomarkers, Prevention & Treatment Cancer Center, and The Key Laboratory of Antibody Technique of Ministry of Health, Nanjing Medical University, Nanjing 210000, China; ^10^Department of Gastrointestinal Surgery, Changzhou No. 2 People's Hospital Affiliated to Nanjing Medical University, Changzhou 213000, China

## Abstract

*Objective.* Rab27b is reported to correlate with cancer development and progression. However, the association between Rab27b expression and the clinical characteristics of colorectal cancer (CRC) is barely investigated. *Methods.* One-step quantitative reverse transcription-polymerase chain reaction (qPCR) test with 18 fresh-frozen CRC samples and immunohistochemistry (IHC) analysis in 113 CRC cases were performed to explore the relationship between Rab27b expression and the clinicopathological features of CRC. Cox regression and Kaplan-Meier survival analyses were executed to evaluate the prognosis of CRC. *Results.* The results demonstrated that the expression levels of Rab27b mRNA and protein were significantly higher in CRC tissues than that in matched noncancerous tissues (*P* < 0.05). Rab27b protein expression in CRC was statistically correlated with serum CEA level (*P* = 0.004), lymph node metastasis (*P* = 0.001), distant metastasis (*P* = 0.009), and TNM stage (*P* = 0.001). Cox multifactor analysis and Kaplan-Meier method suggested that higher Rab27b protein expression (*P* = 0.041) and tumor differentiation (*P* = 0.001) were significantly associated with the overall survival of CRC patients. *Conclusions.* The data indicated that higher expression of Rab27b was observed in CRC tissues and Rab27b may be identified as a useful predictor of metastasis and prognosis for CRC.

## 1. Introduction

Colorectal cancer (CRC) is the third most common malignant tumor worldwide and the second leading cause of cancer mortality in Europe and America [1]. In China, although the incidence of CRC is lower than that in western countries, it has also been growing rapidly in recent years, especially in underdeveloped areas, and CRC occupies the third position in the mortalities caused by cancer [2–4]. The CRC survival significantly correlates with tumor stage at diagnosis and the 5-year survival rate is approximately 65% for CRC patients with lymph node metastasis. Frustratingly, patients with advanced disease with unresectable metastatic lesions suffered a 5-year survival rate of only 5% [5, 6]. Despite various developments in the CRC treatment during the past decades, including improved surgical techniques and novel radiotherapy and chemotherapy strategies, the overall survival rate of patients with CRC has not changed statistically [7]. Moreover, several critical issues, such as rare effective treatment modalities in CRC patients with peritoneal carcinomatosis, resistance of chemotherapy and radiotherapy, and potential adverse effects, remain unsolved [8]. In this regard, it is extremely important to identify specific biomarkers that indicate more accurate clinical characteristics and effective therapeutic targets and predict the prognosis of CRC patients [9, 10].

The Rab family is a type of Ras-like small GTPases that modulate endocytosis and exocytosis vesicle-trafficking control [11, 12]. As soon as Rabs are activated, the vesicles are linked to effectors that are required for vesicle movement, docking, and fusion [13]. Rab27, which is a special member of the small GTPase Rab family and is widely conserved in metazoans, is composed of two isoforms, Rab27a and Rab27b [14, 15]. Rab27b is normally expressed in large number of secretory cells and is believed to play important role in regulating secretory pathways by regulating common Rab27 effectors [16]. Recently, it is reported that aberrant expression of Rab27b contributes to cancer progression and increased Rab27b expression correlates with lymph node metastasis [17, 18]. Rab27b regulates invasive tumor growth of breast cancer cells using and overexpression of the Rab27b indicates poor prognosis in breast cancer (BC) and hepatocellular carcinoma (HCC) [19, 20]. Based on the above studies, Rab27b manifests oncogenic function and plays significant role in cancer development. However, the expression of Rab27b, as well as its clinical characteristics and prognostic significance in CRC, has barely been investigated.

In this study, we detected Rab27b expression in CRC samples as in matched noncancerous samples for comparison by using quantitative real-time polymerase chain reaction (qPCR) and immunohistochemistry (IHC) methods. Moreover, we analyzed the relationship between Rab27b expression and its clinicopathologic features in CRC, especially prognostic significance.

## 2. Materials and Methods

### 2.1. Patients and Tissue Samples

A total of 113 formalin-fixed, paraffin-embedded CRC tissues and matched tumor-adjacent normal tissues were obtained from the Affiliated Hospital of Nantong University from 2006 to 2008. Before surgical therapy, none of the patients had received neoadjuvant chemotherapy, radiation therapy, or immunotherapy. Important clinical data (including gender, age, tumor size, tumor location, histological type, tumor differentiation, serum CEA level, metastasis status, and TNM stage) were collected from each patient's medical records. Clinical staging was performed according to the latest revision of American Joint Committee on Cancer/International Union Against Cancer TNM staging system [21]. Moreover, a panel of 18 fresh CRC tissues and corresponding adjacent noncancerous tissues, obtained from the tissue bank of the Affiliated Hospital of Nantong University, were enrolled in this study simultaneously. Written informed consent was obtained from the patients for publication of this study and any accompanying images. Study protocol was approved by the ethics committee of local hospital.

### 2.2. Detection of the mRNA Expression of Rab27b by One-Step qPCR Test

Total RNA was extracted from 18 cases of the frozen CRC tissues and the matched tumor-adjacent normal tissues using the Trizol reagent (Invitrogen, Carlsbad, CA, USA) according to the manufacturer's guidelines. One-step qPCR analysis was performed with a SensiMixTM One-Step Kit (Quantace, London, UK) using a Real-Time PCR system (Bio-Rad IQ5, Hercules, CA, USA) according to the standard protocol. The primers for Rab27b were as follows: forward primer 5′-TGC GGG ACA AGA GCG GTT CCG-3′ and reverse primer 5′-GCC AGT TCC CGA GCT TGC CGT T-3′. The glyceraldehyde 3-phosphate dehydrogenase (GAPDH) mRNA level was used to standardize the measurements of Rab27b and the primers for GAPDH were as follows: forward primer 5′-TGC ACC ACC AAC TGC TTA GC-3′ and reverse primer 3′-GGC ATG GAC TGT GGT CAT GAG-5′. Total RNA extraction, amplification conditions, and one-step qPCR procedure were described in our previous publication [22].

### 2.3. Detection of the Protein Expression of Rab27b by IHC Analysis

Tissue microarray (TMA) was produced by Alenabio Biotech (Xi'an, China). Core tissue biopsies (2 mm in diameter) were collected from individual paraffin-embedded CRC sections and arranged in the recipient paraffin blocks. The TMA was cut into 4 *μ*m sections and placed on Superfrost charged glass microscope slides. IHC analysis was performed as previously described [22]. TMA sections (4 *μ*m) were deparaffinized in 100% xylene and rehydrated in graded ethanol solutions. Tissue sections were incubated with a primary anti-Rab27b antibody (1 : 100, ab104083, Abcam, Cambridge, UK) in TBS containing 1% bovine serum albumin for 1 hour. After washing, sections were then washed with TBS and incubated with horseradish peroxidase-conjugated anti-rabbit antibody. For the negative control reactions, phosphate-buffered saline was used in place of the primary anti-Rab27b antibody. Rab27b immunostaining was scored by two independent pathologists according to intensity and percentage of positive cells simultaneously. Staining intensity was scored as follows: 0: negative; 1: weakly positive; 2: moderately positive; and 3: strongly positive. The percentage of Rab27b-positive cells was also scored according to 4 categories, in which 1 was given for 0–10%, 2 for 11–50%, 3 for 51–80%, and 4 for 81–100%. The product of the intensity and percentage scores was used as the final staining score. The degree of Rab27b staining was quantified using a two-level grading system as follows: samples with a sum score < 4 were considered as low Rab27b expression while those with a sum score ≥ 4 were considered as high Rab27b expression.

### 2.4. Statistical Analysis

Statistical analyses were performed by using STATA Version 12.0 (Stata Corporation, College Station, TX, USA). The expression of Rab27b mRNA in fresh frozen CRC tissues and in the corresponding noncancerous tissues normalized to GAPDH was analyzed with the Wilcoxon nonparametric signed-rank test. The relationship between Rab27b protein expression and clinicopathological factors was evaluated by chi-square test. Survival rate was estimated by Kaplan-Meier method. Univariate and multivariate analysis was executed using Cox's proportional hazards regression models. For all tests, the significance level for statistical analysis was set at *P* < 0.05.

## 3. Results

### 3.1. Clinical Data of 113 CRC Patients

A total of 113 CRC tissues were collected from 73 men and 40 women and the principal clinical data are summarized in Table 1. The mean age of all patients at the time of surgery was 65.2 years. There were 47 cases with tumor diameter ≥5 cm and 63 with tumor diameter <5 cm. The tumors of 57 cases were located in colon, 52 cases in rectum, and 4 cases in ileocecal junction. The histological type of tumor in the majority of the cases (103 of 113) was adenocarcinoma, whereas the other 10 cases were identified as nonadenocarcinoma type. Only 1 patient suffered from a well-differentiated tumor, 93 had moderate tumor differentiation, and 14 had poor tumor differentiation. There were 12 cases with serum CEA level ≥ 15 ng/mL and 73 cases with serum CEA level < 15 ng/mL. Positive lymph node metastasis was observed in 44 patients while distant metastasis was witnessed in 5 patients. According to TNM staging system, 29 patients were in stage I, 36 patients were in stage II, and 42 patients were in stage III, whereas the remaining 6 patients were in stage IV.

### 3.2. Evaluation of Rab27b mRNA Expression by qPCR Test

**Figure 1:**
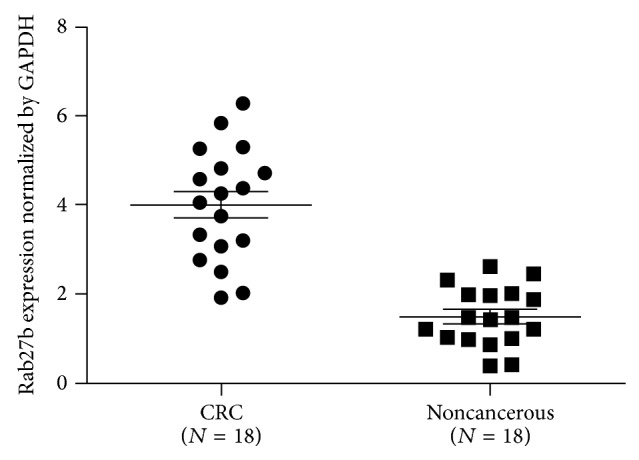
One-step quantitative real-time polymerase chain reaction (qPCR) was employed to evaluate Rab27b mRNA expression levels in 18 colorectal cancer (CRC) tissues compared with matched noncancerous tissues. When normalized to glyceraldehyde 3-phosphate dehydrogenase (GAPDH) mRNA levels, the Rab27b mRNA level in CRC tissues (4.01 ± 0.301) is significantly higher than that in corresponding noncancerous tissues (1.50 ± 0.156).

Total RNA was extracted from 18 CRC tissues and matched tumor-adjacent tissues and subsequently subjected to one-step qPCR for the evaluation of the expression of Rab27b mRNA. When normalized to GAPDH, the means of Rab27b mRNA expression in CRC tissues and the corresponding tumor-adjacent tissues were 4.01 ± 0.301 and 1.50 ± 0.156, respectively (*t* = 7.429, *P* < 0.001). Rab27b mRNA expression averaged 2.6-fold higher in CRC samples than that in noncancerous tissues (Figure 1).

### 3.3. Evaluation of Rab27b Protein Expression by IHC Analysis

**Figure 2:**
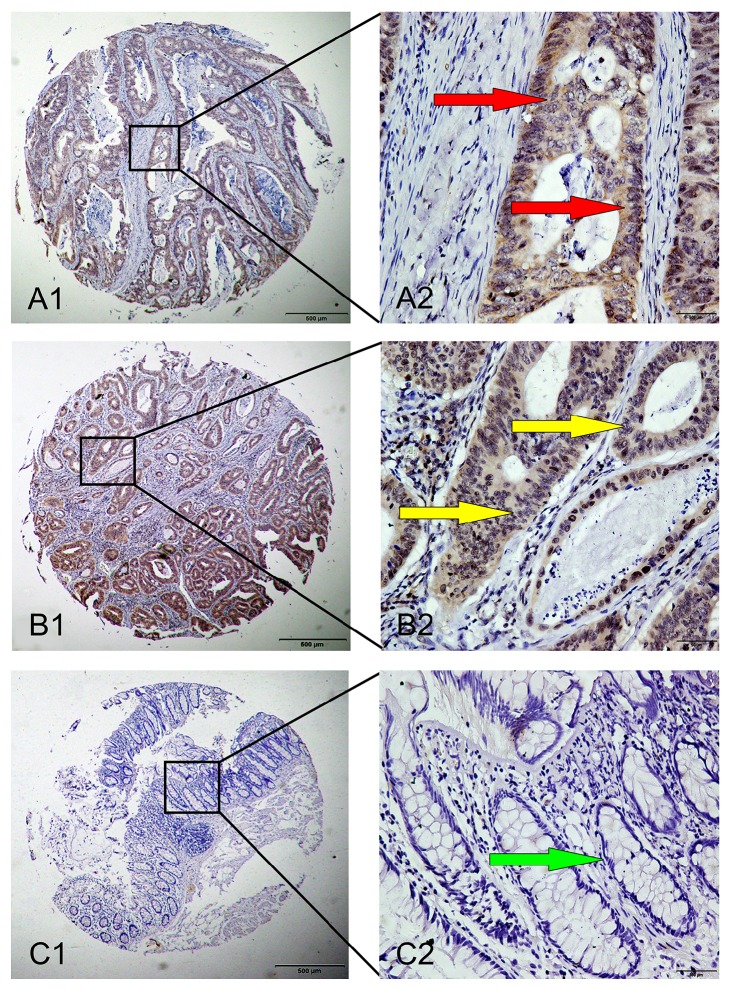
Representative images of Rab27b protein expression in colorectal cancer (CRC) tissues and corresponding noncancerous tissues with tissue microarray (TMA). A1 and A2: positive immunohistochemical (IHC) staining of Rab27b protein in CRC sample. Red arrows show positive staining in the cytoplasm of cancer cells. B1 and B2: positive IHC staining of Rab27b protein in CRC sample. Yellow arrows show positive staining in the nuclei of cancer cells. C1 and C2: negative IHC staining of Rab27b protein in matched noncancerous tissue sample. Green arrow shows negative staining in noncancerous cell (epithelial cell). Original magnification ×40 in A1, B1, and C1; ×400 in A2, B2, and C2.

IHC analysis was performed to explore the Rab27b protein expression in CRC. High Rab27b expression was detected in 49 (43.4%) out of 113 CRC tissues and in 23 (20.4%) out of tumor-adjacent noncancerous tissues. The results showed statistical significance (*P* < 0.05) and were consistent with results of Rab27b mRNA expression. Positive staining was mainly localized in the cytoplasm and nucleus of CRC cells. Typical IHC staining patterns for Rab27b in CRC are shown in Figure 2.

### 3.4. Association between Rab27b Protein Expression and Clinicopathological Items

The relationship between high Rab27b protein expression and important clinicopathological items was shown in Table 1. High Rab27b protein expression was significantly related to serum CEA level (*P* = 0.004), lymph node metastasis (*P* = 0.001), distant metastasis (*P* = 0.009), and TNM stage (*P* = 0.001). In comparison, other clinical items, such as gender, age, tumor size and location, histological type, and tumor differentiation, were barely associated with high Rab27b protein expression (Table 1).

### 3.5. Survival Analysis

**Figure 3:**
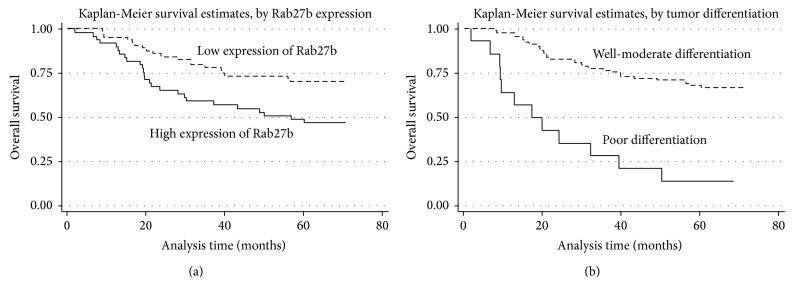
Survival analysis of colorectal cancer (CRC) patients by Kaplan-Meier method. (a) Overall survival rate in patients with high Rab27b protein expression (solid line) was significantly lower than that in patients with low Rab27b expression (dash line). (b) Overall survival rate in patients with poor differentiation of tumor (solid line) was significantly lower than that in patients with well-moderate differentiation of tumor (dash line).

With univariate analysis, the high Rab27b protein expression exhibited a significant correlation with the overall survival rate of 116 CRC patients (*P* = 0.010). In addition, several other clinical items, including tumor differentiation (*P* = 0.001), serum CEA level (*P* = 0.028), distant metastasis (*P* = 0.005), and TNM stage (*P* = 0.002), also showed a statistically significant correlation with the overall survival rate (Table 2). Moreover, all of the above significant factors were enrolled in a further multivariate analysis. The results indicated that high Rab27b expression (*P* = 0.041) and tumor differentiation (*P* = 0.001) are two independent prognostic factors for CRC (Table 2). Kaplan-Meier survival curves indicated that CRC patients with high Rab27b expression and poor tumor differentiation suffered a significantly shorter survival time (Figure 3).

## 4. Discussion

To date, several members of the Rab family have been indicated to act substantially in cancer development. Rab25, for instance, has been demonstrated to inhibit apoptosis as well as promote the proliferation and aggressiveness of ovarian and breast cancer [23, 24]. Rab23 is reported to be overexpressed in gastric cancer and function importantly in tumor invasion [25]. Similarly, Rab27b is described to promote invasive growth and metastasis in estrogen receptor- (ER-) positive cell lines and increased Rab27b expression is associated with poor prognosis in BC patients [17, 19]. Rab27b can also be recognized as a valuable prognostic indicator for HCC patients [20]. How does Rab27b perform oncogenically and facilitate tumorigenesis? Hendrix et al. identified a novel mechanism that illustrates the possible relationships among secretory Rab27b, heat shock protein (Hsp) 90a, and matrix metalloproteinase- (MMP-) 2. Intracellular Hsp90a is secreted into the extracellular environment in a Rab27b-specific manner and MMP-2 activation depends on extracellular Hsp90a chaperoning which is highly associated with Rab27b activity [19]. A great many studies have proved the vital roles of Hsp90a and MMP-2 in tumor invasion and progression [26–28]; hence, it is reasonable to presume the prooncogenic function in cancer. In this present study, we attempted to verify the relationship between Rab27b expression and various clinicopathological attributes of CRC, especially the prognosis significance.

The qPCR test with small samples exhibited a remarkably higher level of Rab27b mRNA expression in CRC tissues than that in noncancerous tissues. Subsequently, we prepared TMA and performed IHC analysis for further evaluation and the data showed a statistically increasing Rab27b protein expression in CRC tissue comparing with that in matched noncancerous tissues. In addition, high Rab27b protein expression was closely correlated with an aggressive phenotype of CRC, including serum CEA level, lymph node metastasis, distant metastasis, and TNM stage. The above data, in agreement with data of the previous study [18, 20], indicate that high level of Rab27b expression correlated to the progressive magnitude of CRC. It is well acknowledged that Rab27b is responsible for several secretory mechanisms and many studies have demonstrated that tumor cells use exosomes to communicate with surrounding tissues and immune cells, developing and maintaining an immunosuppressive microenvironment for tumor progression [29, 30]. Thus, it is rational to propose that Rab27b plays important role in tumor development and is critically associated with tumor progression.

Another intriguing observation that positive expression of Rab27b was detected in stromal cells in the present study deserves to be mentioned. Although it is reported that Rab27b expression was located in the cytoplasm of cancer cells in some previous studies [18, 20], the latest research stated that Rab27b was also expressed in stromal cancer cells with high staining and this result was consistent with our present findings [31].

In addition, it is well known that metastases are severely responsible for most cancer-related mortalities. As for CRC patients, the lymph node metastasis and distant metastasis are two main reasons for the extremely low and desperate 5-year survival rate [5]. In this study, we found that those CRC patients with higher Rab27b expression are prone to have lymph node metastasis and distant metastasis and hence we hypothesize that Rab27b may be identified as a new predictor of metastasis status in CRC.

With regard to the prognostic effect of Rab27b, the univariate and multivariate analysis of Cox regression model showed that CRC patients with elevated Rab27b expression had worse survival outcomes than those expressing lower Rab27b, suggesting the clinical value of Rab27b in predicting the prognosis of CRC patients. Kaplan-Meier analysis also displayed that the life span of CRC patients with high Rab27b expression was notably unfavorable. Our obtained survival data are in line with the previous researches that describe the prognostic role of Rab27b in breast cancer and HCC [19, 20].

To sum up, we demonstrated for the first time that a higher expression of Rab27b was noted in CRC tissues and Rab27b may be utilized as a valuable predictor for positive lymph node metastasis and unfavorable prognosis. Further researches in a larger cohort of CRC are necessary to thoroughly confirm our findings.

## 5. Conclusions

In conclusion, this is the first report of the differential expression of Rab27b in CRC. In this study, we demonstrated high Rab27b expression in CRC tissues and demonstrated that CRC patients with elevated Rab27b expression are prone to encounter tumor metastasis and unfavorable prognosis. Our research is valuable in exploring the characteristics of Rab27b in the development and progression of CRC.

## Figures and Tables

**Table 1:** Correlation of Rab27b expression with clinicopathological characteristics of 113 CRC patients.

Groups	Number	Rab27b		*χ* ^2^	*P* value
		+	%		
Gender					
Male	73	36	49.3	2.98	0.085
Female	40	13	32.5		
Age (years)					
≥60	75	35	46.7	0.99	0.319
<60	38	14	36.8		
Tumor size (cm)					
≥5	47	24	51.1	2.33	0.127
<5	63	23	36.5		
Insufficient data	3	2			
Tumor location					
Colon	57	30	52.6	4.16	0.125
Rectum	52	18	34.6		
Ileocecal junction	4	1	25.0		
Histological type					
Adenocarcinoma	103	43	41.7	1.24	0.266
Nonadenocarcinoma	10	6	60.0		
Tumor differentiation					
Well	1	0	0.0	0.98	0.613
Moderate	93	41	44.1		
Poor	14	7	50.0		
Insufficient data	5	1			
Serum CEA level (ng/mL)					
≥15	12	9	75.0	8.31	0.004^*^
<15	73	23	31.5		
Insufficient data	28	17			
Lymph node metastasis					
Positive	44	28	63.6	12.06	0.001^*^
Negative	69	21	30.4		
Distant metastasis					
Positive	5	5	100.0	6.83	0.009^*^
Negative	108	44	40.7		
TNM stage					
Stages I, II	65	19	29.2	12.44	0.001^*^
Stages III, IV	48	30	62.5		

^*^ *P* < 0.05.

**Table 2:** Univariate and multivariate analysis of prognostic factors in 113 CRC patients for overall survival.

	Univariate analysis			Multivariate analysis		
	HR	*P* > |*z*|	95% CI	HR	*P* > |*z*|	95% CI
Rab27b expression						
High versus low	2.18	0.010^*^	1.204–3.937	2.25	0.041^*^	1.032–4.889
Gender						
Male versus female	1.47	0.239	0.773–2.810			
Age (years)						
≥60 versus <60	1.14	0.681	0.607–2.146			
Tumor size (cm)						
≥5 versus <5	1.23	0.497	0.672–2.267			
Tumor location						
Colon versus rectum versus ileocecal junction	1.46	0.150	0.872–2.461			
Histological type						
Adenocarcinoma versus nonadenocarcinoma	2.37	0.233	0.574–9.778			
Tumor differentiation						
Well and moderate versus poor	0.19	0.001^*^	0.097–0.377	0.17	0.001^*^	0.072–0.378
Serum CEA level (ng/mL)						
≥15 versus <15	2.43	0.028^*^	1.101–5.342	1.62	0.275	0.681–3.862
Lymph node metastasis						
Positive versus negative	1.43	0.233	0.794–2.578			
Distant metastasis						
Positive versus negative	4.40	0.005^*^	1.559–12.407	4.46	0.066	0.908–21.917
TNM stage						
Stage I versus stage II versus stage III versus stage IV	1.75	0.002^*^	1.226–2.502	1.12	0.683	0.647–1.942

^*^ *P* < 0.05.
